# Supplementary material for: Electrochemical grooving of tube inner walls with emphasis on feed strategy and multi-pass effects on material removal and groove geometry
Source: PLoS One. 2025 Sep 8;20(9):e0331972. doi: 10.1371/journal.pone.0331972 (PMC12416709; doi:10.1371/journal.pone.0331972)
Supplement: Attachment — Submitted filename: Response to Reviewers.docx [file pone.0331972.s003.docx]

List of Responses:

**The authors acknowledge the comments of the reviewer and thank them for their comments, advice, and suggestions.**

*Reviewer 1*

The authors have made significant improvements to the paper by addressing the feedback provided by the reviewers, resulting in a clearer presentation of results. Based on these revisions, the paper is now ready for acceptance.

Response to Reviewer 1:

We sincerely thank the reviewer for the encouraging and positive feedback. We greatly appreciate your recognition of the clarity, relevance, and scientific rigor of our study. Your supportive comments are truly motivating and have reinforced the value of our work.

Thank you again for your time and kind evaluation.

**The authors acknowledge the comments of the reviewer and thank them for their comments, advice, and suggestions.**

*Reviewer 2:* Dear author,

Necessary revisions have been performed. Manuscript can be accepted for publishing.

Best wishes.

Response to Reviewer 2:

We sincerely thank the reviewer for the encouraging and positive feedback. We greatly appreciate your recognition of the clarity, relevance, and scientific rigor of our study. Your supportive comments are truly motivating and have reinforced the value of our work.

Thank you again for your time and kind evaluation.

*Reviewer 3*

**The authors acknowledge the comments of the reviewer and thank them for their comments, advice, and suggestions. All the revised parts are highlighted in yellow.**

1. The abstract is too much extended. It is better to shorten the abstract and display only the key findings.

Response 1. We appreciate this valuable comment and have revised the abstract to make it more brief and concise by removing unnecessary explanations (in line ***20*** of page ***2***).

2. The literature survey not only includes the description of the work defined in the previous literature. However, it is the combination of the summary and the critics of each literature. The current literature survey lacks that point. The author should take care of it while writing the introduction.

Response 2. We thank the reviewer for the valuable suggestion regarding the literature survey. In the revised manuscript, we have carefully addressed this concern by not only summarizing the key findings of previous studies but also critically evaluating their strengths and limitations. Specifically, for each cited work, we have added discussion on the parameters or conditions that were not considered, such as electrolyte flow rate, tool feed rate, temperature distribution, and boundary conditions, and we have highlighted how these limitations motivated the present study. These changes have been incorporated throughout the introduction section to ensure a comprehensive and critical literature review. (starting from line ***64*** of page ***3***).

3. Fig. 2 does not show any UEF and HEF conditions.

Response 3. Thank you for your valuable comment. Accordingly, illustrations for the HEF and UEF conditions have been added as Fig. 2d and Fig. 2e, respectively. In-text citations referring to these subfigures have also been included to improve clarity.

4. The initial results showed minimal differences between the outcomes achieved from UEF and HEF conditions. However, the current results show significant differences between the outcomes from UEF and HEF, especially in the entry position as explained in the revised manuscript. How many times have the experiments been repeated? If the results are repeatable, why was the difference not significant in the initial study?

Response 4. The experimental results presented in the revised manuscript are consistent with those reported in the original submission. In the 2^nd^ revision, we have included additional analyses and simulations to provide a clearer explanation of the differences observed between UEF and HEF conditions, particularly at the entry position. The experiments were repeated three times to ensure repeatability, and the outcomes showed high consistency with the initial observations. Since the variations across repetitions were negligible, error bars were not included in the figures. The relevant information can be found in the original manuscript (see ***page 12***, under “Analysis of the Machined Area” section; ***page 15***, under “Unidirectional Electrolyte Flow” section; ***page 18***, under “Conclusions” section in the eighth conclusion). The enhanced analyses, therefore, aim to clarify why significant differences appear in specific regions, without altering the originally reported data.

*NOTE: Equation and figure numbering is listed according to the revised manuscript. The revised section in the main text body are colored according to the response colors shown in this file. The page and line numbers mentioned herein are based on the manuscript version submitted without figures.*

**The authors acknowledge the comments of the reviewer and thank them for their comments, advice, and suggestions.**

*Reviewer 4*

The authors have answered all the questions and improved the paper. The paper can be published in its present form.

Response to reviewer:

We sincerely thank the reviewer for the encouraging and positive feedback. We greatly appreciate your recognition of the clarity, relevance, and scientific rigor of our study. Your supportive comments are truly motivating and have reinforced the value of our work.

Thank you again for your time and kind evaluation.

**The authors acknowledge the comments of the reviewer and thank them for their comments, advice, and suggestions.**

*Reviewer 5*

Authors have now incorporated all the suggestions. The article is now well revised and improved as well.

Response to reviewer:

We sincerely thank the reviewer for the encouraging and positive feedback. We greatly appreciate your recognition of the clarity, relevance, and scientific rigor of our study. Your supportive comments are truly motivating and have reinforced the value of our work.

Thank you again for your time and kind evaluation.

*NOTE: Equation and figure numbering are listed according to the revised manuscript. The revised section in the main text body is colored according to the response colors shown in this file. The page and line numbers mentioned herein are based on the manuscript version submitted without figures.*
